# An Electrode Design Strategy to Minimize Ferroelectric Imprint Effect

**DOI:** 10.1002/advs.70011

**Published:** 2025-05-22

**Authors:** Yu‐Wei Chen, Tung‐Yuan Yu, Chun‐Wei Huang, Tzu‐Hsuan Yu, Yung‐Chi Su, Chao‐Rung Chen, Wei‐Chen Hung, Pei‐Yin Chang, Bhagwati Prasad, Yu‐Chuan Lin, Ramamoorthy Ramesh, Yen‐Lin Huang

**Affiliations:** ^1^ Department of Materials Science and Engineering National Yang Ming Chiao Tung University Hsinchu 300093 Taiwan; ^2^ Taiwan Semiconductor Research Institute Hsinchu 300091 Taiwan; ^3^ Department of Materials Science and Engineering Feng Chia University Taichung 407102 Taiwan; ^4^ Department of Materials Engineering Indian Institute of Science Bangalore Karnataka 560012 India; ^5^ Department of Materials Science and Engineering Rice University Texas 77005 USA; ^6^ Department of Materials Science and Engineering University of California Berkely 94720 USA

**Keywords:** Ferroelectrics, imprint, reliability, retention, ultra‐low‐voltage

## Abstract

The phenomenon of ferroelectric imprint, characterized by an asymmetric polarization switching behavior, poses significant challenges in the reliability and performance of ultra‐low‐voltage ferroelectric devices, including MagnetoElectric Spin‐Orbit devices, Ferroelectric Random‐Access Memory, Ferroelectric Field‐Effect Transistors, and Ferroelectric Tunnel Junctions. In this study, the influence of electrode configuration in different device architectures are systematically investigated on their imprint effect. By tuning the work function of La_0.7_Sr_0.3_MnO_3_ (LSMO) electrodes through oxygen pressure during deposition, precise control over the built‐in voltage offset (V_offset_) in ferroelectric capacitors are demonstrated. This results reveal that higher oxygen pressures increase the work function of LSMO, effectively compensating for V_offset_ and enhancing device stability. Finally, a ferroelectric device with a hybrid bottom electrode of LSMO and SrRuO_3_ is optimized to mitigate the imprint effect. The optimal device showcases small coercive voltage of 0.3 V, a minimal V_offset_ of 0.06 V, excellent endurance (electrical cycle up to 10^9^), and robust zero bias applied polarization retention. These findings provide a practical guideline for electrode design in ferroelectric devices, addressing the imprint effect and improving operational reliability. This approach, combining material tuning and in situ diagnostics, offers a pathway to optimize ferroelectric device performance, with implications for advancing ultra‐low‐power electronics.

## Graphical abstract

A tunable electrode design using La_0.7_Sr_0.3_MnO_3_ enables precise modulation of ferroelectric imprint through oxygen pressure–controlled work function engineering. By applying this strategy to both perovskite and fluorite systems, the built‐in field is effectively suppressed, enabling low‐voltage, high‐endurance ferroelectric devices, including Fe‐FETs. This approach offers a versatile pathway for imprint‐free device integration in next‐generation oxide electronics.

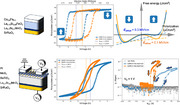

## 1 Introduction

Ferroelectric imprint is a phenomenon observed in ferroelectric materials, characterized by an asymmetry in the polarization switching behavior.^[^ ^1^, ^2^, ^3^ ^]^ This asymmetry often manifests as a voltage shift in the polarization‐voltage (P‐V) hysteresis loop along the applied voltage axis resulting in unequaled coercive voltages. Imprint can be a serious issue in ultralow‐voltage driven ferroelectric devices such as MagnetoElectric Spin‐Orbit devices (MESO),^[^ ^4^, ^5^, ^6^, ^7^, ^8^, ^9^, ^10^, ^11^ ^]^ Ferroelectric Random‐Access Memory,^[^ ^12^, ^13^, ^14^, ^15^ ^]^ Ferroelectric Field Effect Transistor,^[^ ^16^, ^17^, ^18^, ^19^, ^20^, ^21^ ^]^ Ferroelectric Tunnel Junction,^[^ ^22^, ^23^, ^24^ ^]^ and their advanced energy efficient computing applications.^[^ ^25^, ^26^, ^27^ ^]^ This voltage shift can bias one polarization state, causing high operating voltage, data retention issues, and reliability problems over time.^[^ ^28^, ^29^, ^30^ ^]^

Both perovskite‐ and fluorite‐based ferroelectric devices exhibit imprint issues, which limit their potential in commercial applications.^[^ ^1^, ^12^, ^13^, ^14^, ^30^, ^31^ ^]^ This unbalanced ferroelectric polarization state can be caused by several factors, including (1) domain wall pinning induced by interface defects,^[^ ^3^, ^32^, ^33^ ^]^ (2) strain gradient induced electric field along the out‐of‐plan direction described by flexoelectric effect,^[^ ^3^, ^34^, ^35^, ^36^, ^37^, ^38^, ^39^ ^]^ (3) unbalanced top‐to‐bottom electrode contact potential (arguably equivalent to the unbalanced trapped charges at the ferroelectrics‐electrode interfaces),^[^ ^1^, ^32^, ^40^, ^41^ ^]^ and (4) chemical gradient along the out‐of‐plan direction,^[^ ^42^, ^43^ ^]^ can cause non‐uniformities in the local electric field, contributing to imprint. Addressing ferroelectric imprint is crucial for the reliable operation of ferroelectric devices, and researchers work on various strategies, such as optimizing material properties, controlling interfaces, and developing better device structures to mitigate this effect.^[^ ^14^, ^29^ ^]^

Researchers are actively pursuing strategies to mitigate imprint by optimizing material properties, carefully controlling ferroelectric/electrode interfaces, and designing improved device structures. A common approach to minimize the imprint effect in ferroelectrics is to utilize symmetrical top and bottom metallic electrodes to prevent work function mismatches. Additionally, reducing the thickness of the ferroelectric “dead layer” (a non‐switching layer at the electrode interface) and limiting domain wall pinning effects are essential for achieving uniformity and stability in electric field distribution. However, in some particular applications, the selection of top electrode is limited, such as MESO devices and Ferroelectric Field Effect Transistors, the choice of the top electrode is constrained by specific functional requirements, making the mitigation of the imprint effect a particularly challenging issue.

## 2 Results and Discussion

**Figure 1:**
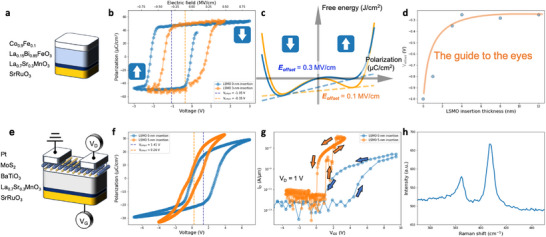
Imprint in the metal‐ferroelectric‐oxide structure. a) Schematic illustration of the magnetoelectric coupling readout film stack. b) P‐V hysteresis loops of Pt(1)/Co_0.9_Fe_0.1_(4)/La_0.15_Bi_0.85_FeO_3_(50)/SrRuO_3_(30) capacitor featured a V_offset_ = −1.05 V (blue dashed line), and Pt(1)/Co_0.9_Fe_0.1_(4)/La_0.15_Bi_0.85_FeO_3_(50)/LSMO(3)/SrRuO_3_(30) capacitor featured a V_offset_ = −0.35 V (orange dashed line), both measured with a 10 kHz triangular voltage waveform. The numbers in parentheses denote the nominal thicknesses in nanometers. The red S‐shaped dashed curve represents the polarization‐voltage dependence derived from Landau free energy. Arrows in c and d indicate the polarization direction. c) Double‐well free energy landscape illustrating the imprint phenomena observed in b, plotted as a function of polarization 𝑃. d) Dependence of V_offset_ on LSMO insertion thickness, varying from the nominal thickness of 0‐nm to 12‐nm. e) Schematic illustration of the ferroelectric field‐effect transistor back‐gate device. The channel length is 40 µm, with the source terminal grounded and the back‐gate bias applied to the bottom electrode. f) P‐V hysteresis loops of Pt(40)/MoS_2_ (1.95)/BaTiO_3_(50)/SrRuO_3_(30) capacitor featured a V_offset_ = −1.41 V (orange dashed line), and Pt(40)/ MoS_2_ (1.95)/BaTiO_3_(50)/LSMO(5)/SrRuO_3_(30) capacitor featured a V_offset_ = −0.26 V (orange dashed line), both measured with a 10 kHz triangular voltage waveform. g) I_D_‐V_GS_ characteristics at room temperature of a representative MoS_2_ under different imprint effect with an on/off ratio >10^5^. The sign of V_GS_ is reversed for consistency with the voltage direction in panels b and f. h) Raman characterization of 3‐monolayer MoS_2_ transferred on BaTiO_3_ surface.

Using the MESO devices as an example, the optimal strategy to harness the ultralow voltage‐driven magnetoelectric effect involves fabricating a heterostructure consisting of a ferromagnetic layer (Co_0.9_Fe_0.1_) and a multiferroic layer (La_0.15_Bi_0.85_FeO_3_), as illustrated in **Figure** **1**a.^[^ ^4^, ^9^, ^10^, ^11^ ^]^ The sample growth and device fabrication details can be found in the method section. We note that an additional challenge in this metal/oxide heterostructure is the oxidation of the Co_0.9_Fe_0.1_ layer, as revealed by the scanning transmission electron microscopy image in Figure S1 (Supporting Information). This interfacial oxidation can degrade the exchange coupling and reduce the efficiency of magnetization switching, ultimately contributing to ferroelectric imprint (as shown in Figure S1b,c, Supporting Information), incomplete polarization switching, and long‐term reliability concerns.^[^ ^44^ ^]^ For the bottom electrode, perovskite SrRuO_3_ is commonly used due to its small lattice mismatch (≈3.93 Å) with the multiferroic layer and its high conductivity (≈180 µΩ×cm).^[^ ^45^ ^]^ Under such a limitation of the top electrode of Co_0.9_Fe_0.1_ and the bottom electrode of SrRuO_3_, the imbalance ferroelectric polarization preference is observed, as depicted by the blue curve in Figure 1b. The V_offset_ indicated by the blue dashed line is calculated by the center voltage of the ferroelectric hysteresis loop. This negatively shifted P‐V loop signifies that the two polarization states have non‐degenerate energies, allowing them to be reversed by an applied voltage but only the polarization‐up state (indicated by the downward arrow in Figure 1b) is stable at zero bias. To better understand this unbalanced switching behavior, we can refer to the Landau‐Devonshire (LD) model. The LD theory describes how the free energy density (F) depends on the order parameter, specifically the ferroelectric polarization (P), in ferroelectric materials. We represent the ferroelectric double‐well energy landscape using a polynomial from LD theory as follows:

(1) $$F=aP^{2}+bP^{4}+cP^{6}+EP$$

where a and b are Landau energy coefficients, F is the free energy density, P is the ferroelectric polarization, and E is the applied electric field. To fit the P‐V curves shown in Figure 1b, we differentiate F with respect to P from Equation (1), resulting in:

(2) $$\frac{dF}{dP}=\frac{V}{d}\;-\;\frac{V_{offset}}{d}=2aP+4bP^{3}+6cP^{5}$$

where d is the ferroelectric layer thickness, and V_offset_ is the fitting parameter to capture the built‐in field. By applying Equation (2) to fit the P‐V loop, we can derive the double‐well free energy curve that represents our observed asymmetric ferroelectric switching behaviors, as illustrated by the blue line in Figure 1c. The tilted double‐well free energy landscape indicates that there is only one stable polarization state, where the free energy is minimized below zero. This stable state corresponds to the upward polarization direction, maintained by a built‐in field strength of approximately −0.3 MV cm^−1^.

Given the constraints in selecting the top electrode, we focus on modifying the bottom electrode to reduce the built‐in voltage across the La_0.15_Bi_0.85_FeO_3_ layer. Inspired by the study of Pu Yu et al., which shows that atomically precise control of La_0.7_Sr_0.3_MnO_3_ (LSMO) terminations can lead to distinct polarization preference states in ferroelectrics,^[^ ^46^ ^]^ and building on our previous work where different bottom oxide electrodes altered the as‐grown polarization direction in BiFeO_3_ across three crystal orientations,^[^ ^47^ ^]^ we aim to explore similar strategies. Specifically, we incorporate LSMO​ into the bottom electrode design. We note that the chemical composition of LSMO is La_0.7_Sr_0.3_MnO_3_, representing the nominal stoichiometry; however, the actual film stoichiometry is influenced by the growth conditions, which will be discussed in detail later. For the sake of simplicity, we use LSMO to present the material utilized in optimizing 𝑉_offset_. The orange curve in Figure 1b displays a more symmetric P‐V hysteresis loop (with V_offset_ = −0.35 V) compared to the blue curve. This was achieved by inserting a 3‐nm layer of LSMO between the SrRuO_3_ and La_0.15_Bi_0.85_FeO_3_​ layers. The corresponding double‐well free energy profile, depicted by the orange curve in Figure 1c and derived using Equations (1) and (2), indicates the achievement of bistable polarization states with this 3‐nm LSMO insertion. Figure 1d summarizes the LSMO insertion layer thickness dependence to the V_offset_, which are saturated around −0.3 V above 4‐nm. The reasons we utilize hybrid 30‐nm SrRuO_3_ and 3‐nm LSMO are considering the lattice mismatch is smaller for SrRuO_3_ to La_0.15_Bi_0.85_FeO_3_ and the single layer of LSMO is typically much more electrically resistive resulting in larger RC delay. We note that the LSMO insertion layers used here were deposited by pulsed laser deposition without the assistance of reflection high‐energy electron diffraction and the without substrate termination control. The nominal thickness is calibrated by X‐ray reflectivity and cross‐sectional TEM images.

Another example of ferroelectric imprint arising from asymmetric electrode constraints is observed in ferroelectric field‐effect transistors (Fe‐FETs). In a Fe‐FET, a ferroelectric insulator acts as the gate dielectric in a metal‐oxide‐semiconductor field‐effect transistor (MOSFET), where the polarization state of the ferroelectric gate modulates the channel conductance, enabling non‐destructive data readout.^[^ ^15^, ^17^, ^18^, ^19^, ^20^, ^21^ ^]^ This device architecture inherently features one oxide/metal interface and one oxide/semiconductor interface, both of which are essential and cannot be substituted. To demonstrate our LSMO insertion approach is generic phenomena and applicable, we fabricated the Ferroelectric Field Effect Transistor as illustrated in Figure 1e. Three layers of MoS_2_ membranes were transferred onto the surface of 100‐nm BaTiO_3_, either with or without a 5‐nm LSMO insertion layer between the SrRuO_3_ and BaTiO_3_, which were grown on a single‐crystal SrTiO_3_ (001) substrate. The source and drain terminals were fabricated using 40‐nm Pt, with a channel length of 40 µm. Figure 1f presents representative ferroelectric hysteresis loops. The blue curve displays imbalanced ferroelectric switching with a V_offset_ = 1.41 V. In contrast, the orange curve, representing the device with an LSMO insertion layer, exhibits more symmetric switching with a V_offset_ = 0.26 V. Notably, in the Pt/MoS_2_/BaTiO_3_/SrRuO_3_ configuration, the ferroelectric polarizations tend to shift downward (toward the positive bias in the P‐V measurements). This behavior can be attributed to the compressive strain typically present in ferroelectric films. As the strain gradually relaxes from the bottom to the surface, it creates a strain gradient across the film, resulting in a polarization pinning effect.^[^ ^19^, ^38^, ^48^ ^]^ Figure 1g demonstrates the switching characteristics of Pt/MoS_2_/BaTiO_3_/(LSMO)/SrRuO_3_ Fe‐FETs. The I_D_‐V_GS_ curves were measured by sweeping the back‐gate voltage at V_D_ = 1 V, with V_S_ connected to the ground. The curves exhibit a clear clockwise hysteresis and a voltage‐shifted loop, mimicking the ferroelectric switching behavior of BaTiO_3_. A high on/ off ratio of over 10^5^ indicates a high‐quality ferroelectric/semiconductor interface. With the insertion of a 5‐nm LSMO layer, the orange curve shows a lower threshold voltage, consistent with the P‐V measurements shown in Figure 1f. Figure 1h shows the Raman spectrum measured on the MoS_2_ transferred onto BaTiO_3_ surface excited by 488 nm line in air ambient environment.

**Figure 2:**
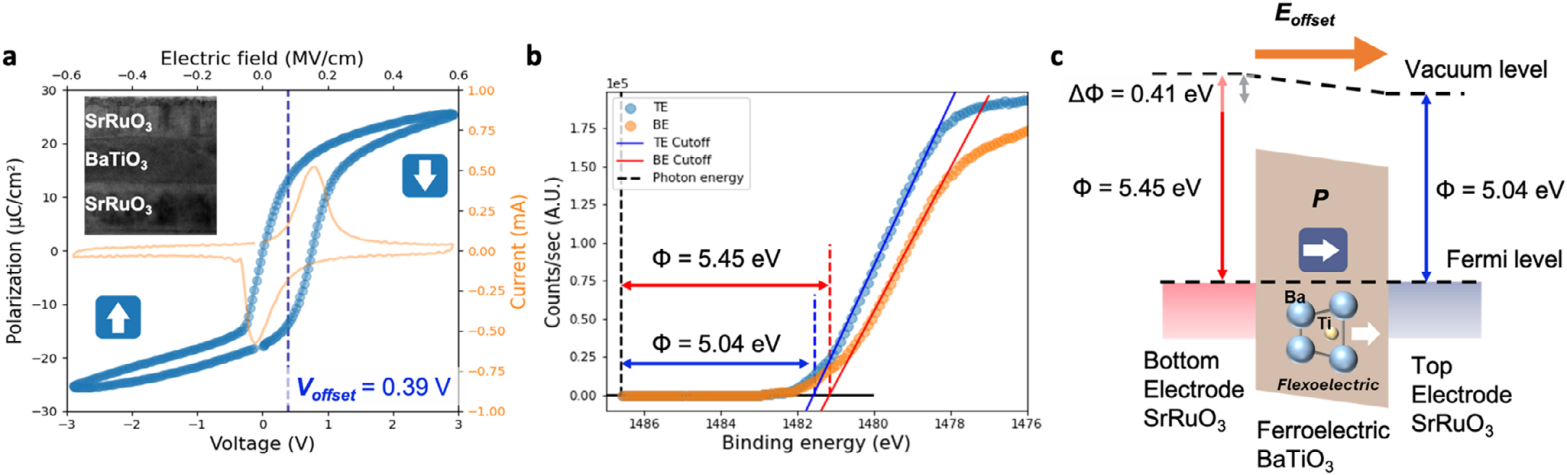
Imprint in the symmetric metal‐ferroelectric‐metal structure. a) P‐V (blue curve) and I‐V (orange curve) hysteresis loops of symmetric electrodes (SrRuO_3_ 30‐nm) ferroelectric (BaTiO_3_ 50‐nm) capacitor measured with a 10 kHz triangular voltage waveform. The V_offset_ indicated by the dash line is 0.39 V. The inset image is cross‐section TEM image of the test structure. b) High binding energy scan (cutoff) measured by XPS for the top (blue) and bottom (orange) electrodes SrRuO_3_ with a photon energy 1486.6 eV. The intersects of cutoff edges and zero indicate the work function of the electrodes. c) Schematic illustration of the band alignment cross the top electrode (ϕ = 5.04 eV), the ferroelectric (flexoelectric effect), and the bottom electrode (ϕ = 5.45 eV).

After optimizing the asymmetrical electrodes and demonstrating the ability to tune the V_offset_ through the insertion of LSMO, we began to explore whether it is possible to overcome the inherent imprint effect in ferroelectrics. Conventionally, researchers have employed symmetrical top and bottom metallic electrodes to minimize imprint effects. However, despite the use of such symmetrical electrodes, V_offset_ in ferroelectric P‐V(E) hysteresis loops are still commonly observed across various studies.^[^ ^49^, ^50^, ^51^, ^52^ ^]^ Here we fabricated an epaxial ferroelectric capacitor test system with symmetrical top and bottom metallic electrodes, SrRuO_3_(30)/BaTiO_3_(50)/SrRuO_3_(30) as shown in the inset of **Figure** **2**a, to mitigate abovementioned causes for imprint issue. The numbers in parentheses denote the nominal thicknesses in nanometers. Figure 2a shows a typical ferroelectric P‐V hysteresis loop with a shifted V_offset_ = 0.39 V ($V_{C}^{-}$ = −0.01 V, $V_{C}^{+}$ = +0.77 V), which is consistent to the I‐V hysteresis loop shown in orange curve, for the symmetrical MFM (SrRuO_3_(30)/BaTiO_3_(50)/SrRuO_3_(30)) capacitors. Compared to the asymmetry electrode case shown in Figure 1b,f, the symmetrical top and bottom SrRuO_3_ electrodes indeed reduce the V_offset_. To further unveil unbalanced ferroelectric polarization switching mechanism, we proceeded to examine the work function of the top and bottom layer of SrRuO_3_ difference by X‐ray Photoemission Microscopy (XPS). Figure 2b shows the XPS spectra obtained from the top and bottom SrRuO_3_ layer with photon energy of 1486.6 eV and in situ Ar sputtering. The work function of the top SrRuO_3_ layer is measured to be 5.04 eV, which is lower than the bottom SrRuO_3_ layer. measured to be meaning. This reduction in work function is likely due to the lower crystallinity of the top layer, resulting from strain relaxation as shown in Figure S2 (Supporting Information). Figure 2c depicts the energy band diagram constructed from the work functions of the top and bottom SrRuO_3_ layers. The ferroelectric polarization is pointing toward the bottom SrRuO_3_ layer reflecting the positive V_offset_ measured in Figure 2a. We model this preference polarization state as a function of temperature to mimic the film cooling process. Figure S3a (Supporting Information) illustrates how the polarization evolves according to the Landau‐Devonshire (LD) energy equation, showing an increase in polarization as the temperature drops below the Curie temperature (T_C_). The phase‐field simulations reveal that the average polarization, computed as the mean value across the 50 × 50 discrete grid points, exhibits a preferred direction in the presence of an internal electric field induced by V_offset_. This internal field biases the energy landscape, leading to a dominant polarization direction. Conversely, when no internal field is applied, the average polarization is reduced due to the symmetric energy landscape, resulting in balanced polarization states across the grid as shown in Figure S3b (Supporting Information).

**Figure 3:**
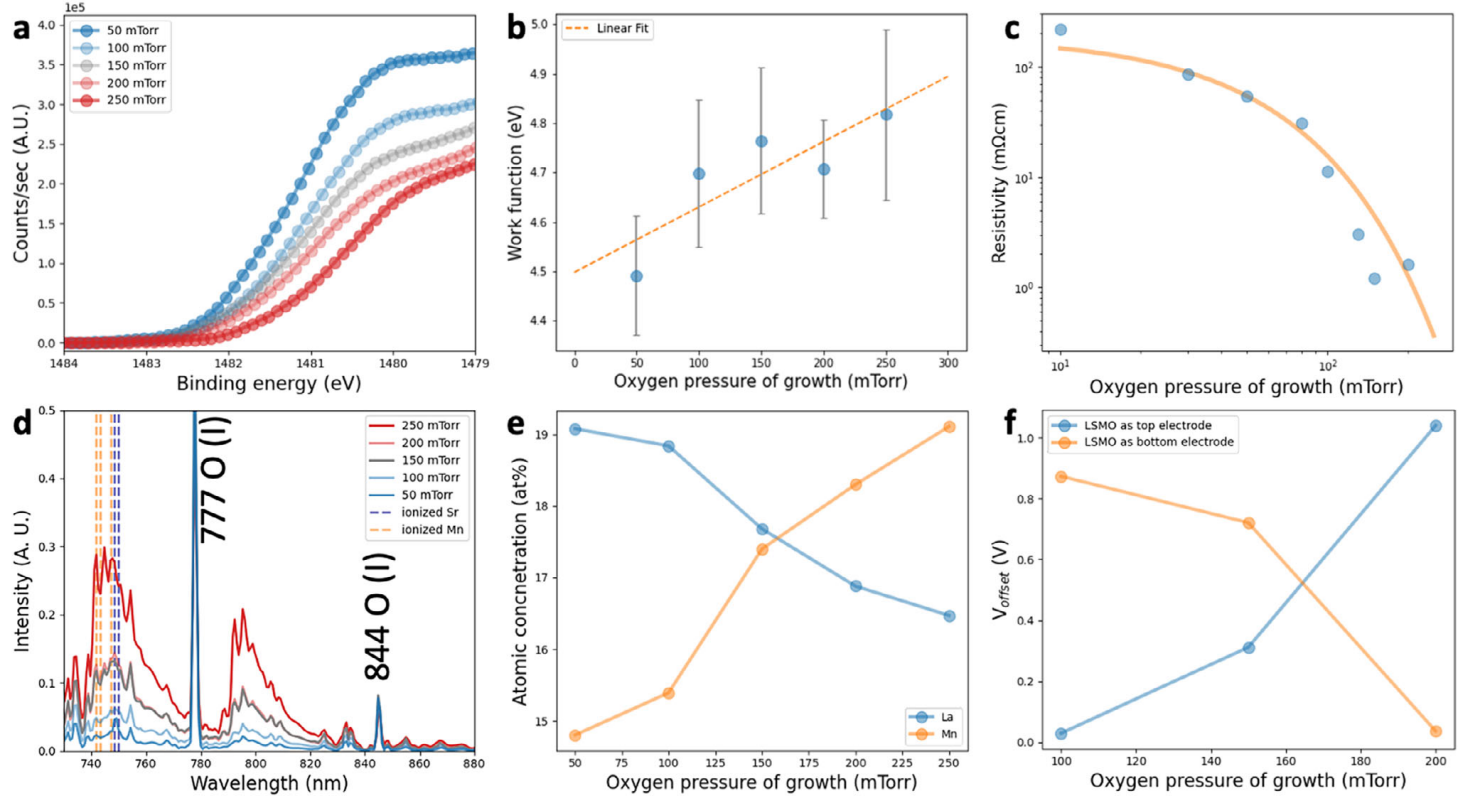
The tunability of La_0.7_Sr_0.3_MnO_3_ via different growth pressure. a) Work function measurements obtained via XPS for LSMO thin films grown under varying oxygen pressures during growth (50–250 mTorr), indicating shifts in work function values as a function of pressure. b) Extracted work function of LSMO thin films grown under varying oxygen pressures during growth. c) Dependence of LSMO thin films resistivity on oxygen pressures during g. d) Optical emission spectra under different oxygen growth pressures, with peaks corresponding to ionized Sr and Mn, marked in blue and orange dash lines. The most prominent two peaks are contributed from oxygen (I) located at 777 and 844 nm.^[^ ^56^ ^]^ e) Elemental stoichiometry of La and Mn as a function of electrode configuration, contrasting LSMO as top and bottom electrodes, highlighting pressure‐dependent compositional variation. f) V_offset_ as a function of LSMO thin films grown under varying oxygen pressures during growth with reversed stacking sequence of LSMO electrodes. The blue (orange) curve indicates the V_offset_ measured in the ferroelectric capacitor with LSMO as the top (bottom) electrode.

Having demonstrated the strong correlation between the unavoidable V_offset_ in ferroelectric capacitors with symmetrical top and bottom electrodes and the mismatch in their electrode work functions, we next focus on compensating for this V_offset_ ​ by leveraging the tunability of the LSMO layer. To explore the feasibility of making the ferroelectric capacitor devices with a minimal V_offset_, we conducted the work function measurements on the LSMO films fabricated under various oxygen pressures (50 – 250 mTorr) at 700 °C by pulsed laser deposition as shown in **Figure** **3**a. Figure 3b displays the extracted work functions from the XPS spectra, revealing a monotonic increase in work function with rising oxygen pressure, ranging from approximately 4.3 to 5.0 eV. In contrast, the work function of SrRuO_3_ remains unaffected by growth oxygen pressure, as shown in Figure S4 (Supporting Information). Additionally, we measured the resistivity of LSMO films as a function of growth oxygen pressure, presented in Figure 3c. The results show an exponential dependence of resistivity on oxygen pressure: films grown at lower oxygen pressures exhibit highly insulating behavior, which inhibits ferroelectric switching.

After observed the strong influence of oxygen growth pressure to the LSMO films, we utilize optical emission spectroscopy (OES) to monitor the plasma plume during pulsed laser deposition. In the interaction between the plume and given deposition oxygen pressure, dissociative processes are expected to produce highly reactive oxygen atoms, driving the oxidation reactions within the plume.^[^ ^53^, ^54^ ^]^ Using OES, we can detect subtle differences in the optical emission spectrum in real time under different growth conditions. Figure 3d displays the optical emission spectra within the 730 – 800 nm range (full spectra are provided in the Figure S5, Supporting Information). Notably, these spectra reveal distinct features, particularly at the emission peaks of ionized Sr and Mn, highlighted by the blue and orange dashed lines, respectively, in Figure 3d. With increasing oxygen pressure, the intensity of Mn emission peaks clearly rises. This suggests that oxygen concentration affects the cation population, with Mn cation emission showing a monotonic increase in the oxygen pressure range of 50 – 250 mTorr. Figure 3e summarizes the atomic percentages of La and Mn measured by XPS (detailed analysis is available in the Figures S6 and S7, Supporting Information), which align with the trends observed in the optical emission spectra. The Mn concentration increases with higher oxygen pressure, reinforcing the theoretical calculation that B‐site termination in perovskite structures can exhibit higher work functions as shown in Figure S8 (Supporting Information).^[^ ^55^ ^]^ This observation is consistent with the work function results shown in Figure 3a,b. Moreover, the XPS results reveal that the work function differences are indeed surface‐related, as evidenced by the variations observed with different Ar‐ion sputtering durations (Figure S15, Supporting Information). This further strengthens the argument that the work function modulation originates from surface termination regulation. To demonstrate the capability to manipulate the V_offset_ in ferroelectric capacitor by LSMO electrodes, we fabricated test structures of LSMO (30, bottom electrode highlighted in orange)/BaTiO_3_(50)/SrRuO_3_(30) and SrRuO_3_(30)/BaTiO_3_(50)/LSMO(30, top electrode highlighted in blue) with 100, 150, and 200 mTorr LSMO growth oxygen pressures as shown in Figure 3f. When LSMO is used as the top electrode, the BaTiO_3_ layer remains fully strained. In this case, the flexoelectric effect contributes to the imprint, and when combined with the work function difference between the electrodes, these effects effectively compensate each other—resulting in a V_offset_ ≈0 V at an LSMO growth pressure of 100 mTorr. In contrast, when LSMO is used as the bottom electrode, the BaTiO_3_ layer becomes partially relaxed, as shown in Figure S7 (Supporting Information). In this relaxed state, the flexoelectric contribution is reduced, and the imprint is primarily governed by the work function difference between the electrodes. Consequently, a larger imprint field is observed, yielding a V_offset_ ≈0.9 V at the same LSMO growth pressure. We note that the chemical gradient can be ruled out for inducing the V_offset_ in our case supported by the TEM/EDS measurements as shown in Figure S10 (Supporting Information).

Furthermore, the imprint effect is a widely observed phenomenon in ferroelectric materials. To demonstrate the generality of this behavior, we also investigated a fluorite‐structured ferroelectric, Hf_0.5_Zr_0.5_O_2_, and observed a similar offset voltage trend. This behavior is similarly attributed to the asymmetry in electrode work functions, underscoring the universality of this effect across different ferroelectric systems, as presented in Figures S11 and S12 (Supporting Information).

**Figure 4:**
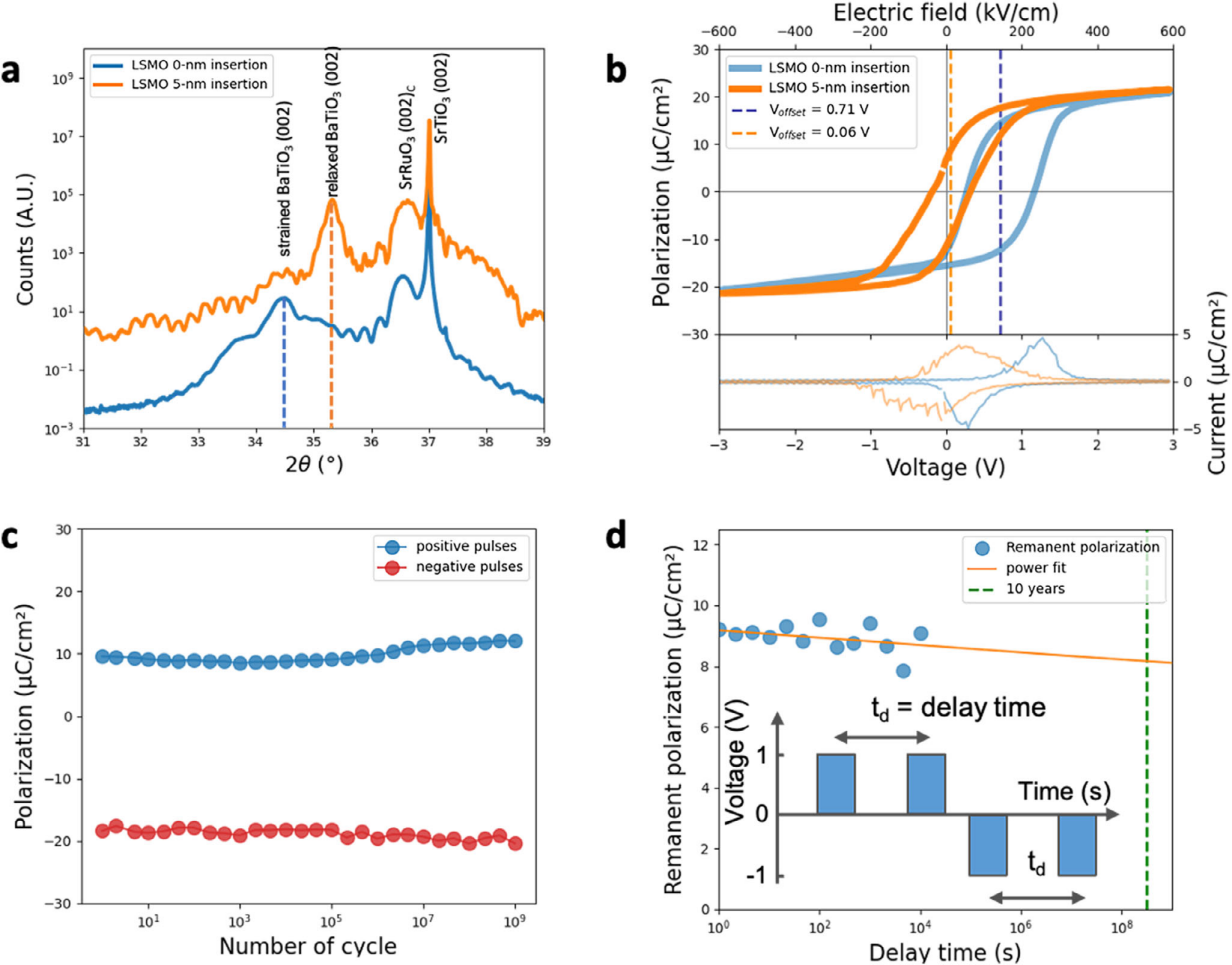
Minimized V_offset_ ferroelectric switching. a) X‐ray theta‐2theta scan of (002) peaks from the SrRuO_3_(30)/LSMO(5)/BaTiO_3_(50)/SrRuO_3_(30) heterostructure grown on the (001) SrTiO_3_ single crystal substrate. The C in the SrRuO_3_ (002)_C_ is denoted as cubic coordination system. b) P‐V hysteresis loops of SrRuO_3_(30)/LSMO(5)/BaTiO_3_(50)/SrRuO_3_(30) capacitor shows a 𝑉_offset_ = 0.06 V (highlighted in orange dashed line). On the other hand, the SrRuO_3_(30)/BaTiO_3_(50)/SrRuO_3_(30) capacitor featured a V_offset_ = 0.71 V (highlighted in blue dashed line), both measured with a 10 kHz triangular voltage waveform. c) Positive (blue) and negative (red) remanent polarization as a function of number of switching cycles of SrRuO_3_(30)/LSMO(5)/BaTiO_3_(50)/SrRuO_3_(30) capacitor. d) Retention property characterized by remanent polarization (*P_r_*) as a function of delay time (*t_d_*) between the write and read pulses. The inset illustrates the *P_r_* readout protocol. A 1 V write pulse, exceeding V_C_ (as shown in b), is applied with a pulse width of 1 s to set the polarization direction. Following this, a read pulse of the same polarity is applied after the delay time *t_d_*. The remanent polarization is calculated as $P_{r}=\int_{0}^{t_{p}}\left(i_{write}-i_{read}\right)dt\;$, where *i_write_* and *i_read_* are the current measured during the write and read pulses, respectively, and *t_p_* is the pulse width.

With this understanding of LSMO, we revisit the feasibility of achieving ultralow coercive voltage and minimal imprint V_offset_ ferroelectric capacitor for practical applications. **Figure** **4**a shows X‐ray θ‐2θ scans around the 002 Bragg peak for representative films from the heterostructures of SrRuO_3_(30)/BaTiO_3_(50)/SrRuO_3_(30) with (shown in orange line) and without the 5‐nm LSMO (shown in blue line) insertion. Figure 4b shows the ferroelectric properties measured from the film stack of BaTiO_3_ with 5‐nm LSMO grown in 200 mTorr oxygen pressure and insertion hybrid bottom electrode highlighted in orange curve. With this optimized device, we investigated the fatigue and retention characteristics of optimal test capacitors using a pulsed measurement setup. During the fatigue experiments, the capacitors were subjected to square bipolar pulses with frequencies ranging from 1 kHz to 20 kHz and amplitudes between +2 V and +5 V. Periodic interruptions in the cycling allowed for pulse measurements of the switched and non‐switched polarization values in both directions, utilizing the same 2‐ms triangular pulse train employed for polarization testing. Figure 4c presents the results for a test conducted at +2 V and 1 MHz, demonstrating minimal fatigue after 10^9^ cycles. Fatigue tests performed at lower frequencies and higher cycling voltages up to +5 V exhibited similarly robust behavior, underscoring the durability of these capacitors. As a reference, the SrRuO_3_(30)/BaTiO_3_(50)/SrRuO_3_(30) device shows two stable polarization states only when a dc +1.05 V (minimal) applied as shown in Figure S13 (Supporting Information).

The retention measurements evaluate the capacitor's ability to switch polarization states after being written into a specific logic state and the extent of polarization loss due to aging in that state. Figure 4d presents data from an experiment where the capacitor was written with a −3 V, 1 ms‐wide pulse and read using a two‐set pulse with a 1 V amplitude. The results demonstrate a significant signal difference of 10 – 8 µC cm^−2^ between the switched and non‐switched polarization states throughout the testing period. Additionally, logic state retention experiments were conducted to confirm the capacitors’ capability to reliably store and distinguish between two logic states. These measurements show a polarization difference exceeding 10 µC cm^−2^ between the two states, sustained for at least 10^4^ seconds. Extrapolated to 10‐year data retention shown in green dash line in Figure 4d, the remanent polarization still has the value of ≈8 µC cm^−2^. A significant advantage of this retention measurement is that no bias needs to be applied during remanent polarization characterization, attributed to the balanced ferroelectric switching and minimized imprint effect, as shown in Figure 4b. This feature showcases the robustness and reliability of the device for long‐term memory applications.

## 3 Conclusion

In conclusion, we address the persistent challenge of ferroelectric imprint—a phenomenon characterized by asymmetric polarization switching that undermines the performance and reliability of ferroelectric devices, particularly in low‐voltage applications such as MESO devices and FeRAM. By leveraging the tunability of LSMO electrodes, we present an electrode design strategy to mitigate imprint and enhance device performance. Our study demonstrates that precise control of LSMO work function through oxygen pressure during pulsed laser deposition enables effective compensation of the built‐in V_offset_, achieving nearly symmetric ferroelectric switching with ultralow coercive voltages (V_C_ ≈0.3 V and V_offset_ = 0.06 V). The optimized ferroelectric capacitors exhibit excellent durability, negligible fatigue after 10^9^ cycles, and outstanding data retention exceeding 10 years with remanent polarization values maintained at ≈8 µC cm^−2^. Moreover, the minimized imprint effect eliminates the need for applied bias during retention characterization, showcasing the robust reliability of these devices. This comprehensive approach, integrating electrode design, material tuning, and in situ diagnostics, paves the way for next‐generation ferroelectric devices with exceptional stability, energy efficiency, and long‐term performance.

## 4 Experimental Section

### Synthesis of Materials Using Pulsed‐Laser Deposition

Bottom electrode layers/ferroelectric layer/top electrode layer heterostructures were grown on SrTiO_3_ (001) substrates via pulsed‐laser deposition. All depositions were performed at a typical laser fluence of 1.0–1.5 J cm^−2^ using a KrF excimer laser (248 nm). The SrRuO_3_ top and bottom electrodes were grown at a temperature of 700 °C and an oxygen pressure of 100 mTorr. The LSMO insertion layers were grown at a temperature of 700 °C and an oxygen pressure of 50 – 250 mTorr. Optical emission spectroscopy (OES) was employed to in‐situ monitor the plasma plume dynamics of LSMO during the deposition process. The ferroelectric layers, BaTiO_3_, were grown at a temperature of 650 °C and an oxygen pressure of 20 – 50 mTorr. The other ferroelectric layers, La_0.15_Bi_0.85_FeO_3_, were grown at a temperature of 700 °C and an oxygen pressure of 100 mTorr on DyScO_3_ (110) single crystal substrates. Following growth, the samples were cooled in 500 Torr of oxygen to room temperature to promote oxidation.

### Synthesis of Materials using DC Magnetron Sputtering

Co_0.9_Fe_0.1_ layers were deposited under a 200 Oe magnetic field to align the magnetic easy axis of the free layer parallel to that of the pinned layer in contact with the La_0.15_Bi_0.85_FeO_3_ at room temperature and 5 mTorr of Ar. To prevent oxidation of the underlying layers, the devices were capped with a 2.5 nm Pt layer.

### X‐ray Photoelectron Spectroscopy (XPS) Measurements

X‐ray Photoelectron Spectroscopy (XPS) measurements were conducted using a Thermo Fisher Scientific Theta Probe equipped with a monochromatic Al K_𝛼_ X‐ray source (1486.6 eV). The base pressure in the analysis chamber was maintained at 10^−9^ Torr to minimize contamination. Survey and high‐resolution spectra were acquired to identify elemental compositions and analyze chemical states. In situ Ar^+^ ion sputtering was performed to remove surface contaminants and access subsurface information. The work functions of the top and bottom electrodes were determined using XPS with in situ Ar sputtering to ensure accurate and contamination‐free measurements. For the top SrRuO_3_ electrode, XPS measurements were conducted after removing surface contamination. The C 1s peak was used as an indicator to monitor surface cleanliness. Once the C 1s signal was completely eliminated through Ar sputtering, the work function spectrum of the top electrode was acquired. To measure the work function of the bottom SrRuO_3_ electrode, depth profiling was performed using Ar etching in the XPS chamber. During the depth profiling process, the elemental composition to identify the transition is carefully monitored to the bottom SrRuO_3_ layer. The absence of residual Ba signals from the BaTiO_3_ ferroelectric layer confirmed the clean interface, allowing them to proceed with acquiring the work function spectrum of the bottom electrode. This approach ensured accurate work function measurements by minimizing contamination and verifying the integrity of the electrode interfaces. All measurements were performed at room temperature, and the data were corrected for possible charging effects by adjusting the binding energy scale accordingly. Quantitative analysis was conducted based on relative sensitivity factors for each element. flexoelectric.

### Device fabrication

Ferroelectric capacitor devices were patterned using UV lithography with a mask aligner at the Nano Facility Center in National Yang Ming Chiao Tung University. The typical ferroelectric capacitor devices featured diameter of 50 – 5 µm. The etching process was carried out using Ar‐ion sputtering in a high‐vacuum chamber with a typical etching rate of 1 nm min^−1^.

### Ferroelectric Hysteresis Loop Measurements

Polarization‐electric field hysteresis loops were measured using a TF 3000 analyzer (aixACCT Systems GmbH) at 1 kHz with a double‐bipolar waveform. The waveform amplitude was varied between 1 and 3 V, depending on specific measurement requirements. Tungsten probe tips (Everbeing Int'l Corp. model: T20‐10) with a tip radius of 1 µm were used to establish electrical contact with the sample electrodes.

### Polarization Fatigue Measurements

Ferroelectric fatigue measurements were carried out by TF 3000 analyzer (aixACCT Systems GmbH). A successive bipolar triangular voltage waves of 20 kHz were applied on the test devices, followed by a 1 kHz bipolar field to characterize the P‐E loops.

### Polarization Retention Characterization

Retention measurements were conducted using a TF 3000 analyzer (aixACCT Systems GmbH). A preset voltage (3 V for 1 ms) was applied at the beginning of each cycle to pre‐pole the polarization in the device under test. No measurement was performed during this preset pulse. A two‐set pulse with a 1 V amplitude was then applied to measure the remanent polarization calculated from the switching current.

### Structure Characterizations

The crystal structures of the prepared samples were characterized using synchrotron‐based X‐ray diffraction with photon energy of 10 keV at the TLS beamline 17B of the National Synchrotron Radiation Research Center (NSRRC), Taiwan. The sample cross section imaging and corresponding elemental distributions were investigated using field emission scanning electron microscopy (FESEM, JEOL JSM‐6700F) equipped with energy‐dispersive X‐ray spectroscopy (EDS). Microstructural analysis was further conducted using high‐resolution transmission electron microscopy (HRTEM, JEOL JEM‐2010) operated at 200 kV.

## Conflict of Interest

The authors declare no conflict of interest.

## Author Contributions

Y.L.H. designed, directed the study, analyzed results, and wrote the manuscript. Y.W.C. grew the materials, fabricated ferroelectric devices, conducted measurements including XRD, ferroelectric test, AFM, and XPS. Y.W.C. aided in the data analysis. T.Y.Y. performed the XPS measurements. C.W.H. performed TEM characterization. Y.C.S, W.C.H, P.Y.C. aided in grew the materials and performed ferroelectric test. C.R.C performed the OES measurements. B.P. fabricated the ME devices. Y.W.C., T.Y.Y., C.W.H., Y.C.S, W.C.H, P.Y.C., B.P., R.R. and Y.L.H. all made contributions to writing the manuscript.

## Supporting information



Supporting Information

## Data Availability

The data that support the findings of this study are available from the corresponding author upon reasonable request.
