# Supplementary material for: An Electrode Design Strategy to Minimize Ferroelectric Imprint Effect
Source: Adv Sci (Weinh). 2025 May 22;12(29):e70011. doi: 10.1002/advs.70011 (PMC12362776; doi:10.1002/advs.70011)
Supplement: Supplementary file 1 — Supporting Information [file ADVS-12-e70011-s001.docx]

**Supplementary Materials for**

An Electrode Design Strategy to Minimize Ferroelectric Imprint Effect

Yu-Wei Chen^1^, Tung-Yuan Yu^2^, Chun-Wei Huang^3^, Tzu-Hsuan Yu^1^_,_ Yung-Chi Su^1^, Chao-Rung Chen^1^, Wei-Chen Hung^1^, Pei-Yin Chang^1^, Bhagwati Prasad^4^, Yu-Chuan Lin^1^, Ramamoorthy Ramesh^5,6^, Yen-Lin Huang^1*^

1. *Department of Materials Science and Engineering, National Yang Ming Chiao Tung University, Hsinchu, Taiwan.*
2. *Taiwan Semiconductor Research Institute, Hsinchu, Taiwan.*
3. *Department of Materials Science and Engineering, Feng Chia University, Taichung, Taiwan.*
4. *Department of Materials Engineering, Indian Institute of Science, Bangalore, Karnataka, India*
5. *Department of Materials Science and Engineering, Rice University, Texas, USA.*
6. *Department of Materials Science and Engineering, University of California, Berkely, USA.*

*e-mail: [yenlinhuang@nycu.edu.tw](mailto:yenlinhuang@nycu.edu.tw)

**S1. Interface oxidation induced ferroelectric imprint.**


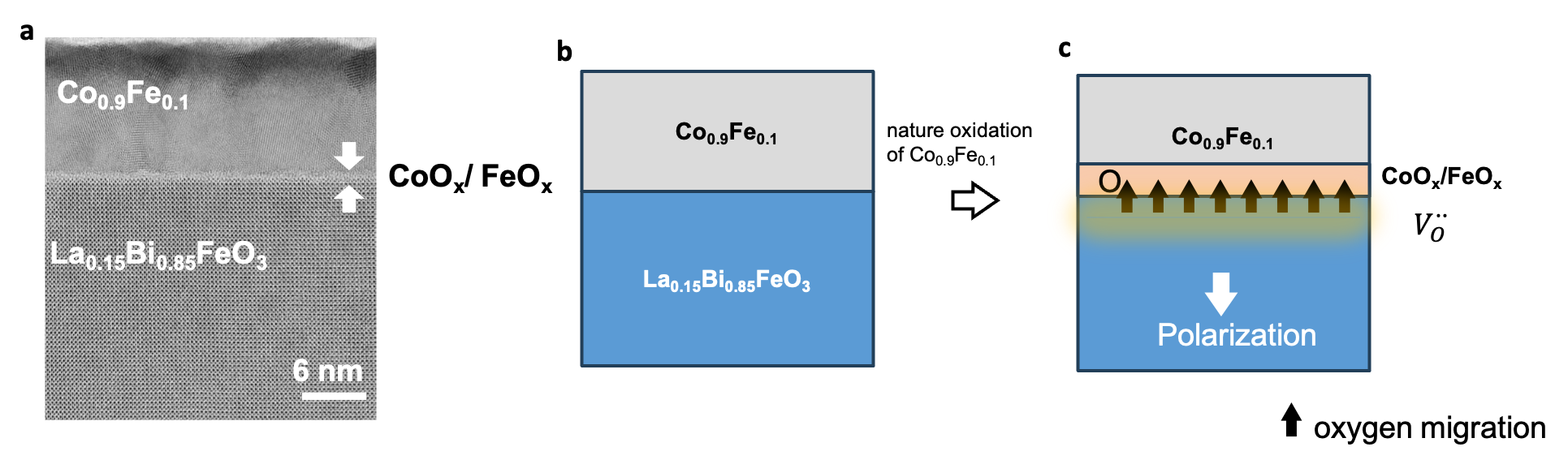

**a,** Cross-sectional STEM image showing the interface between the ferromagnetic layer, Co_0.9_Fe_0.1_ and the multiferroic layer, La_0.15_Bi_0.85_FeO_3_. **b,** Schematic illustration depicting the natural oxidation of the Co_0.9_Fe_0.1_ layer, leading to the formation of an oxidized interfacial layer. **c,** Schematic representation of oxygen vacancy generation at the interface, resulting in a built-in polarization that inherently points downward.

**S2. High-resolution TEM analysis of the interfaces between the top and bottom SrRuO_3_ electrodes and the BaTiO_3_ layer.**

**
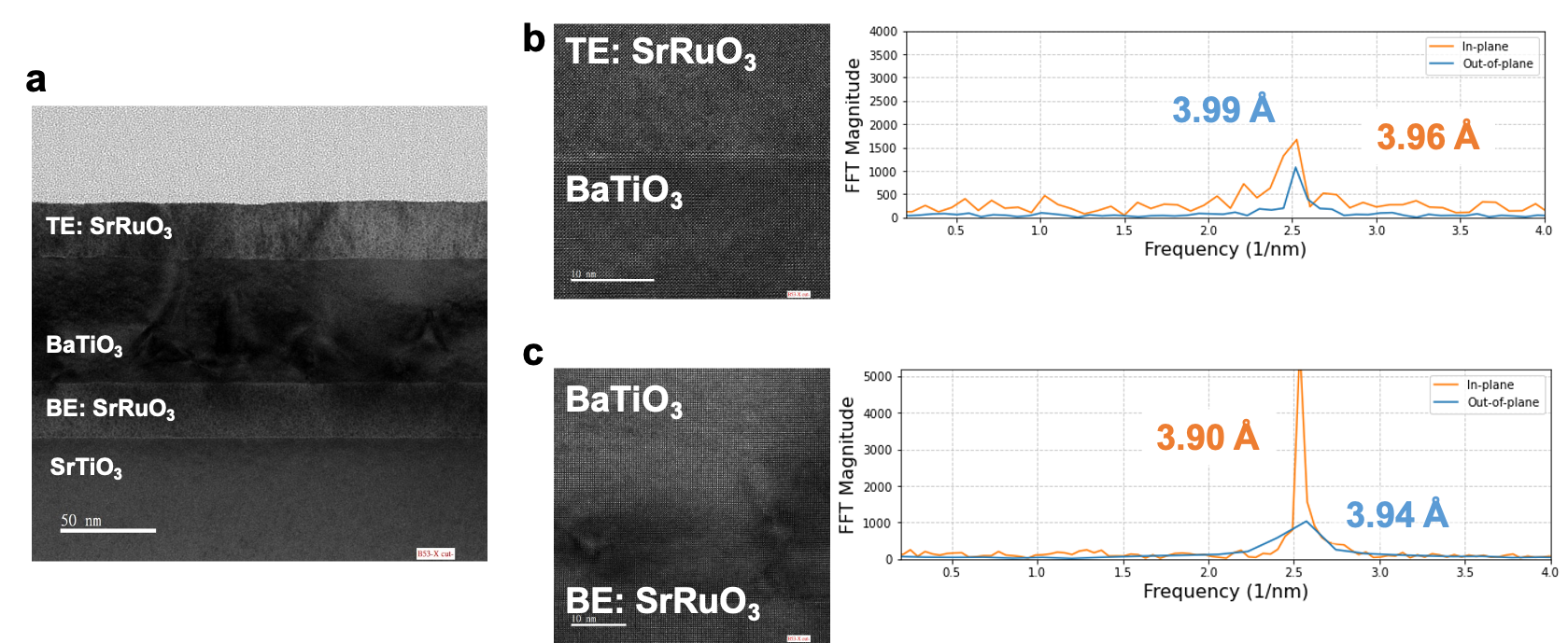
**

**a,** Low-magnification TEM image showing the full cross-sectional stack: SrRuO_3_ (top electrode)/BaTiO_3_/SrRuO_3_ (bottom electrode) grown on a SrTiO_3_ substrate. **b,** High-resolution TEM image of the interface between the top SrRuO_3_ electrode and BaTiO_3_, along with the extracted in-plane and out-of-plane lattice constants. The in-plane lattice constant of 3.96 Å is close to the relaxed SrRuO₃ bulk value, indicating strain relaxation in the top electrode. **c,** High-resolution TEM image of the interface between the bottom SrRuO_3_ electrode and BaTiO_3_, showing an in-plane lattice constant of 3.90 Å, which is closely matching that of the SrTiO_3_ substrate indicating that the bottom SrRuO_3_ layer remains strained.

**Phase-field simulations**. In the phase-field simulation, the polarization field served as the order parameter for describing ferroelectric materials. The temporal evolution of the polarization field was governed by the time-dependent Ginzburg-Landau (TDGL) equation,
$\frac{\partial P_{i}(r,t)}{\partial t}=-L\frac{\delta F_{p}}{\delta P_{i}\left( r,t \right)} (i=1,2,3)$,

where 𝑡 represents time, 𝑟 denotes the spatial position, $P_{i}\left( r,t \right)$ is the polarization component, 𝐿 is the kinetic coefficient, and $F_{p}$​ is the total free energy of the system, expressed as:

$F_{p}=\iiint(f_{bulk}+f_{elastic}+f_{elec}+f_{grad})dV$,

where 𝑉 represents the system volume, $f_{bulk}$ is the Landau bulk energy density, $f_{elastic}$​ is the elastic energy density, $f_{elec}$ is the electrostatic energy density, and $f_{grad}$​ is the gradient energy density. The bulk energy density, $f_{bulk}$, is described by a sixth-order polynomial in terms of the polarization, given by:

$f_{bulk}=\alpha_{1}\left( P_{1}^{2}+P_{2}^{2}+P_{3}^{2} \right)+\alpha_{11}\left( P_{1}^{4}+P_{2}^{4}+P_{3}^{4} \right)+\alpha_{11}\left( P_{1}^{2}P_{2}^{2}+P_{1}^{2}P_{3}^{2}+P_{2}^{2}P_{3}^{2} \right)+\alpha_{112}\left[ P_{1}^{4}\left( P_{2}^{2}+P_{3}^{2} \right)+P_{2}^{4}\left( P_{1}^{2}+P_{3}^{2} \right)+P_{3}^{4}\left( P_{1}^{2}+P_{2}^{2} \right) \right]+\alpha_{111}\left( P_{1}^{6}+P_{2}^{6}+P_{3}^{6} \right)+\alpha_{123}P_{1}^{2}P_{2}^{2}P_{3}^{2}$,

where $P_{1}$, $P_{2}$, and $P_{3}$​ represent the polarization components, and $\alpha_{1}$, $\alpha_{11}$, $\alpha_{12}$, $\alpha_{111}$, $\alpha_{112}$, and $\alpha_{123}$​ are the Landau energy coefficients. Among these coefficients, only$\alpha_{1}$​ is temperature-dependent and follows the Curie-Weiss law, expressed as:

$\alpha_{1}=(T-T_{C})/2\varepsilon_{0}C_{0}$*,*

where $T_{C}$ is the Curie temperature, $C_{0}$ is the Curie constant, and $\varepsilon_{0}$ is the vacuum permittivity. The 2-D gradient energy can be expressed as:

$f_{grad}=\frac{1}{2}\kappa(\frac{\partial^{2}P_{1}}{\partial x^{2}}+\frac{\partial^{2}P_{1}}{\partial y^{2}})$*,*

where $\kappa$ is the gradient energy coefficient. The electrostatic energy density responsive to the built-in field is expressed as:

$$f_{elec}=-E_{built-in}\cdot P_{1}$$

For simplicity, we only consider the polarization in one-dimension ($P_{1}$) and zero elastic deformation ($f_{elastic}=0$). The Curie temperature is set to be 375 K for thin film BaTiO_3_.

**S3. Phase-field simulations of temperature dependence of ferroelectric polarization.**

**
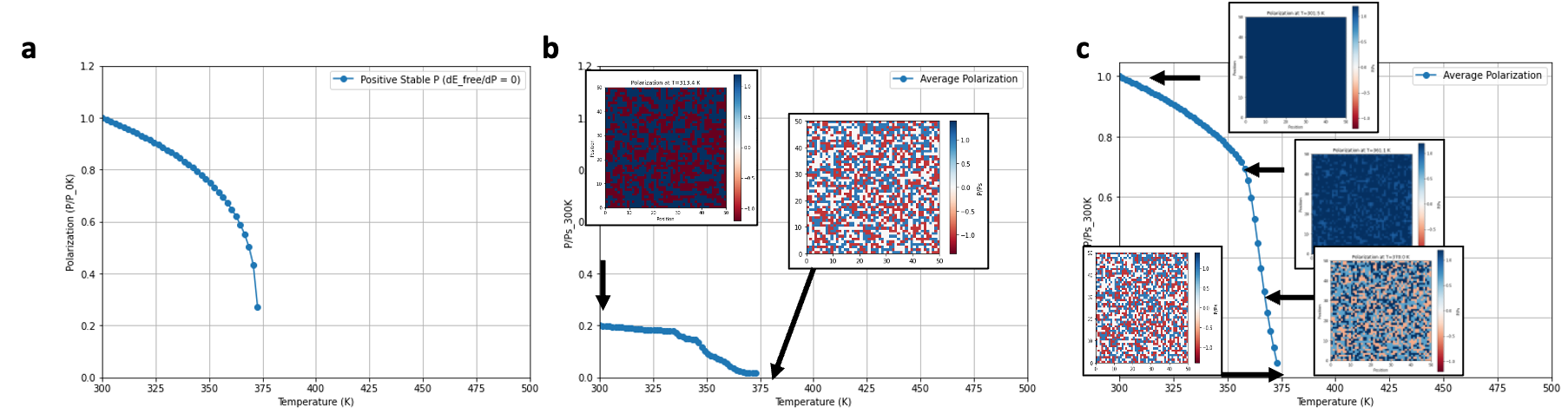
**

**a,** The positive stable polarization states described by the L-D equation are plotted as a function of temperature, illustrating the gradual reduction in stable polarization as the temperature approaches the Curie point. **b,** the spatial distribution of polarization is shown at selected temperatures, with polarization maps revealing the evolution from a uniformly polarized state at low temperatures to a mixed state during the cooling process. The initial polarization state is set to be randomly distributed ranging from (-P, +P) in the 50 by 50 grids. **c,** the average polarization is plotted as a function of temperature, with a build-in field of 180 kV/cm, demonstrating the progressive polarization and reduce domain disorder at lower temperatures. The polarization maps highlight the preference polarization direction induced by the build-in field.

**S4. Work function measurements obtained via XPS for SrRuO_3_ thin films grown under varying oxygen pressures during growth (50-250 mTorr).**


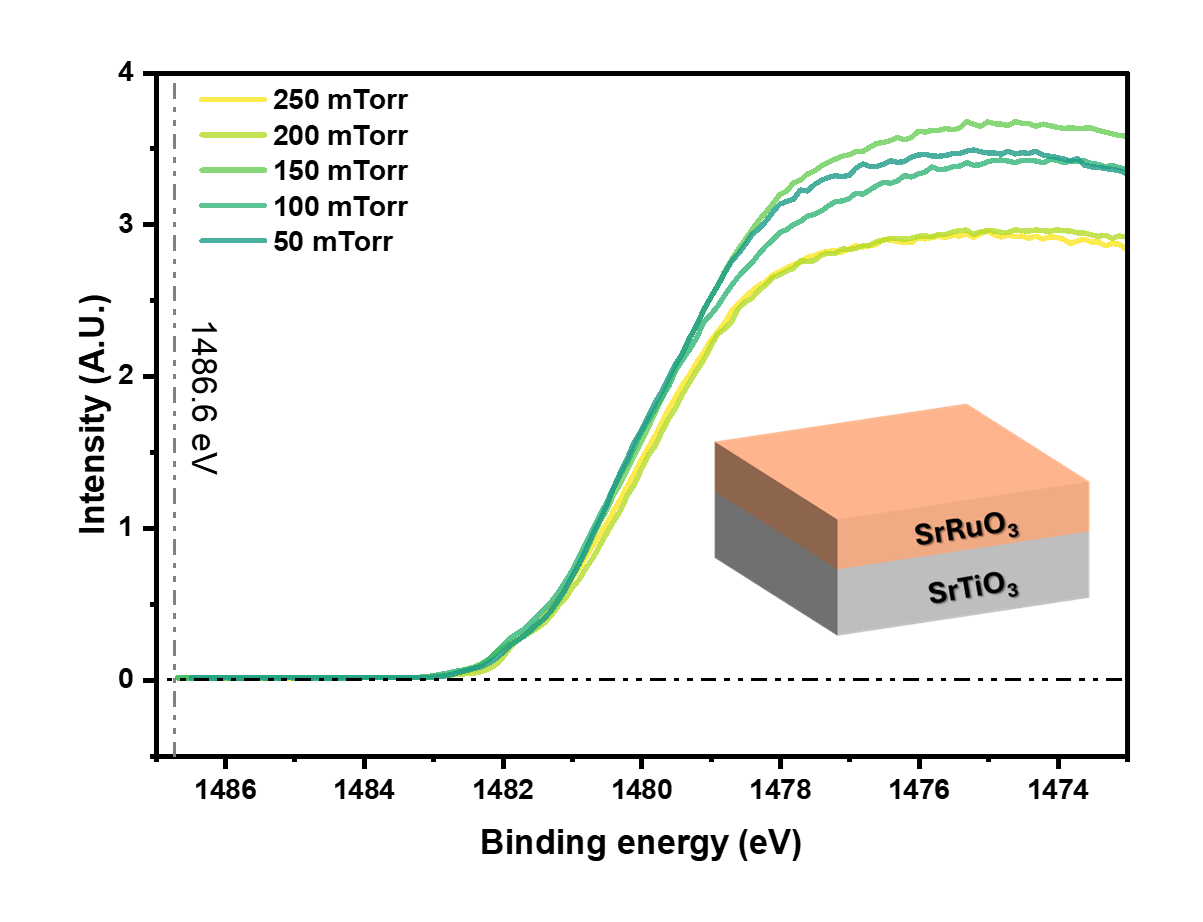


**S5. Optical emission spectra of LSMO under different oxygen pressures.**

**
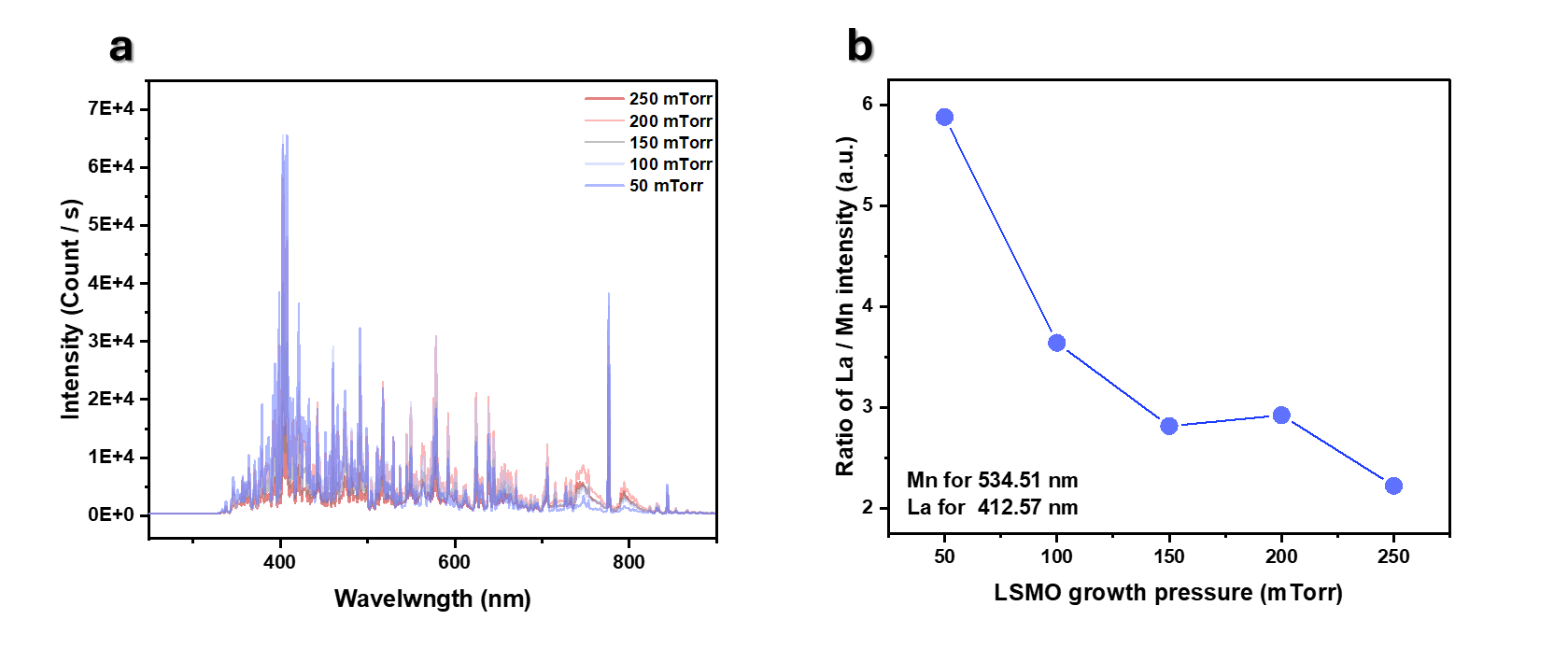
**

**a,** Optical emission spectra acquired during the growth of LSMO films under varying oxygen pressures (50, 100, 150, 200, and 250 mTorr). **b,** The intensity ratio of La to Mn emission peaks, extracted from their respective characteristic wavelengths at 534 nm (La) and 412 nm (Mn), as a function of oxygen pressure.

**S6. Detailed XPS analysis.**

**
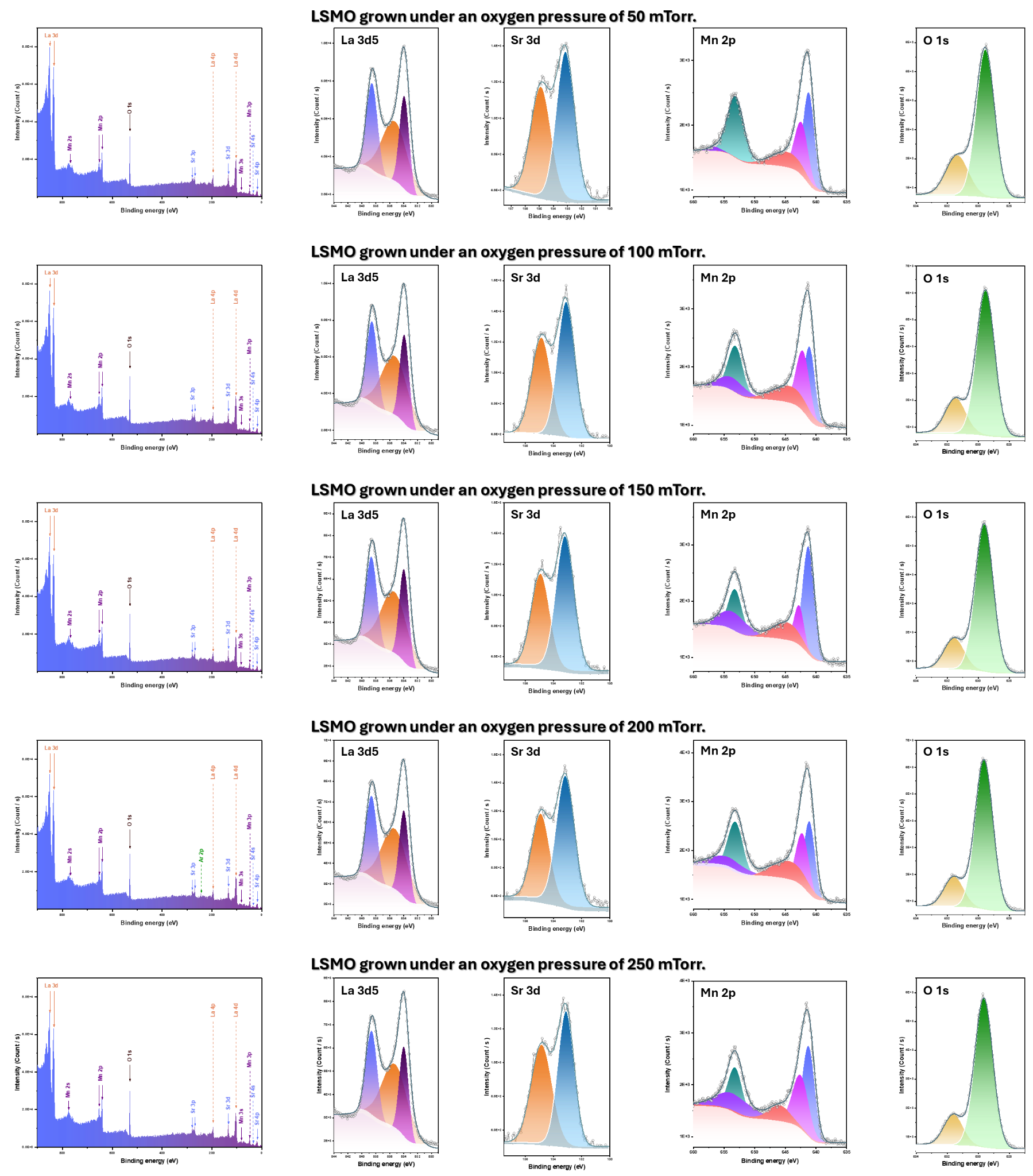
**

The elemental composition of LSMO under varying oxygen growth pressures was analyzed using XPS measurements. The oxygen and strontium concentrations remain relatively constant at approximately 60 at% and 6 at%, respectively, regardless of the background conditions. In contrast, the concentrations of manganese (Mn) and lanthanum (La) exhibit opposing trends with increasing oxygen pressure: Mn content increases, while La content decreases.

**S7. Composition of elements in LSMO films as a function of growth pressure.**

**
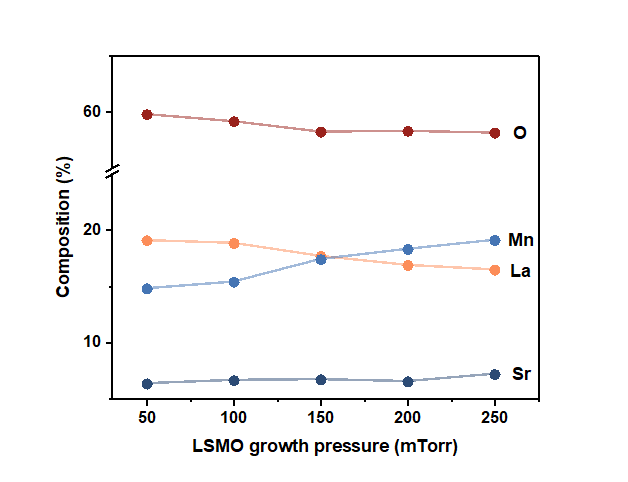
**

The concentrations of oxygen (O) and strontium (Sr) remain constant at approximately 60 at% and 6 at%, respectively, across all pressures. In contrast, manganese (Mn) content increases while lanthanum (La) content decreases as the growth pressure increases.

**S8. Schematic of surface element-dependent work function in LSMO (La_0.67_Sr_0.33_MnO_3_).**
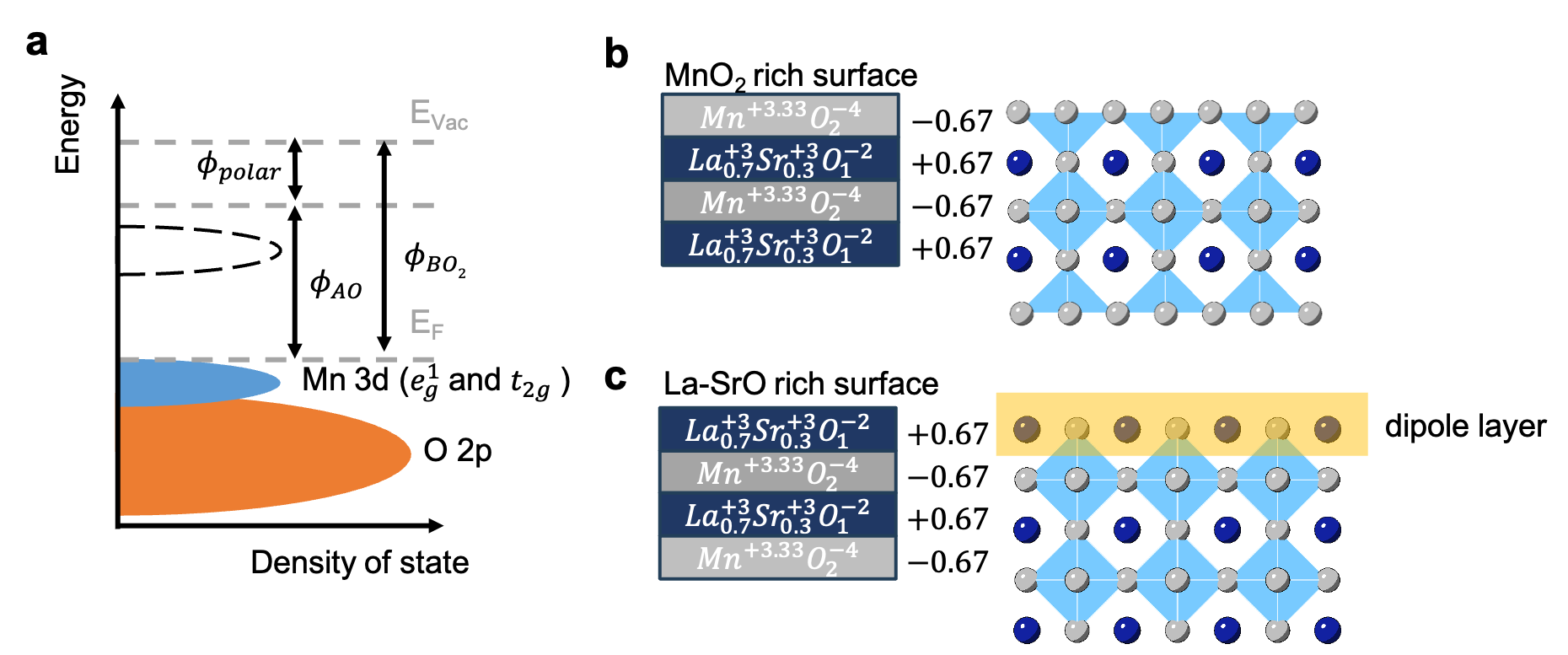


**a,** Energy level diagrams showing the shift in vacuum level (E_vac_) relative to the Fermi level (E_F_) for MnO₂-terminated (top) and (La,Sr)O-terminated (bottom) LSMO surfaces. The AO-terminated surface exhibits a lower work function due to a surface dipole pointing outward. **b,** Layer-resolved stoichiometry and formal charges associated with each termination, highlighting the positive surface dipole in AO termination and the negative dipole in BO₂ termination. **c,** The AO-terminated surface reduces the energy barrier for electron emission, leading to a lower work function compared to the BO_2_-terminated case. Remade from reference[1].

**S9. High-resolution TEM analysis of the interfaces between the top and bottom SrRuO_3_ electrodes and the BaTiO_3_ layer.**
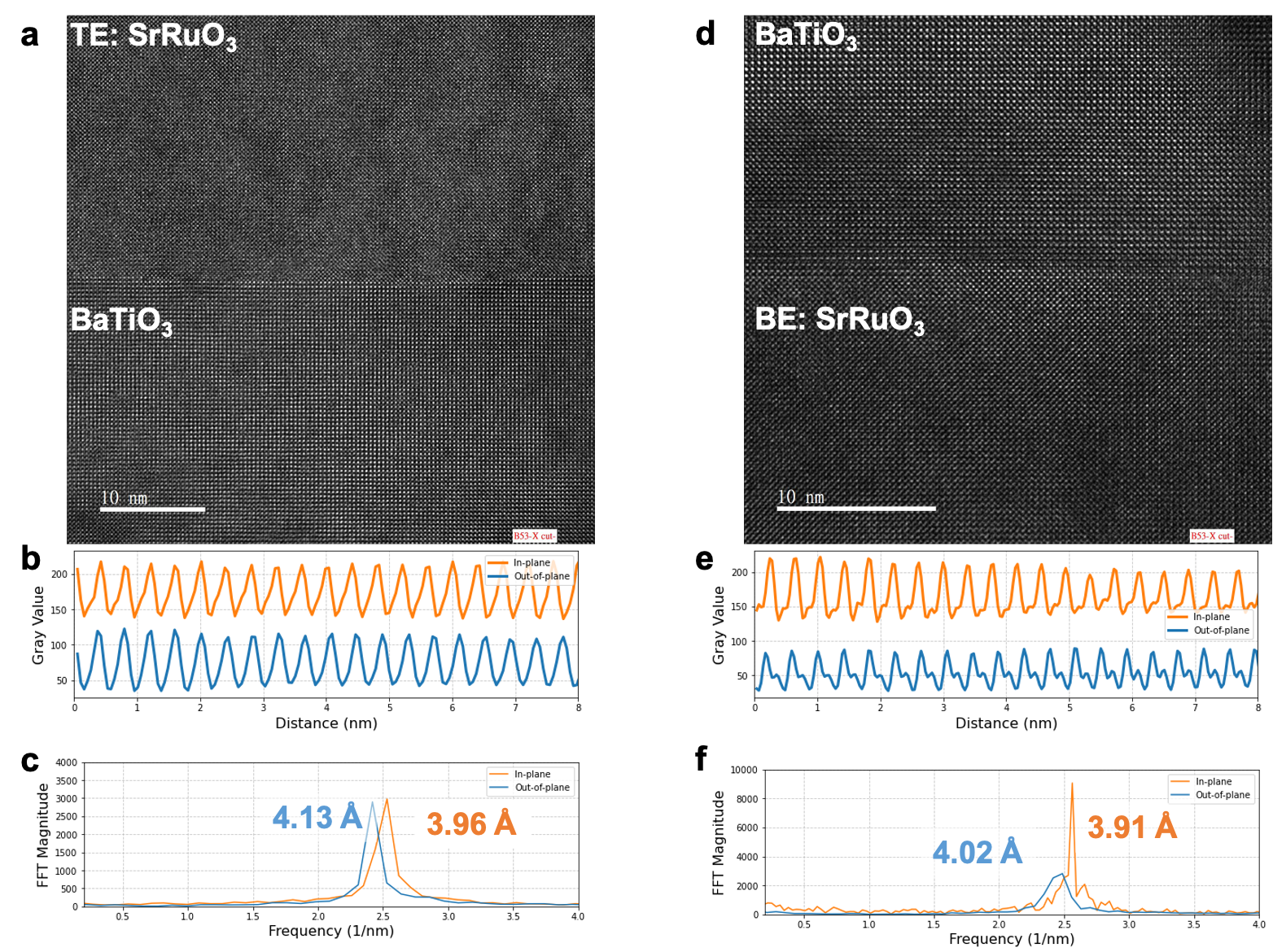


**a,** High-resolution TEM image showing the interface between the top SrRuO_3_ electrode and BaTiO_3_ for the case of LSMO served as bottom electrode. **b,** Corresponding line profiles of the in-plane (orange) and out-of-plane (blue) lattice parameters extracted from the image in **a**. **c,** Fast Fourier Transform (FFT) analysis of the lattice parameters presented in **b**. **d,** High-resolution TEM image showing the interface between the bottom SrRuO_3_ electrode and BaTiO_3_ for the case of LSMO served as top electrode. **e,** Corresponding line profiles of in-plane (orange) and out-of-plane (blue) lattice parameters extracted from the image in **d**. **f,** FFT analysis of the lattice parameters presented in **e**.

**S10. BaTiO_3_** **film composition analysis.**

**
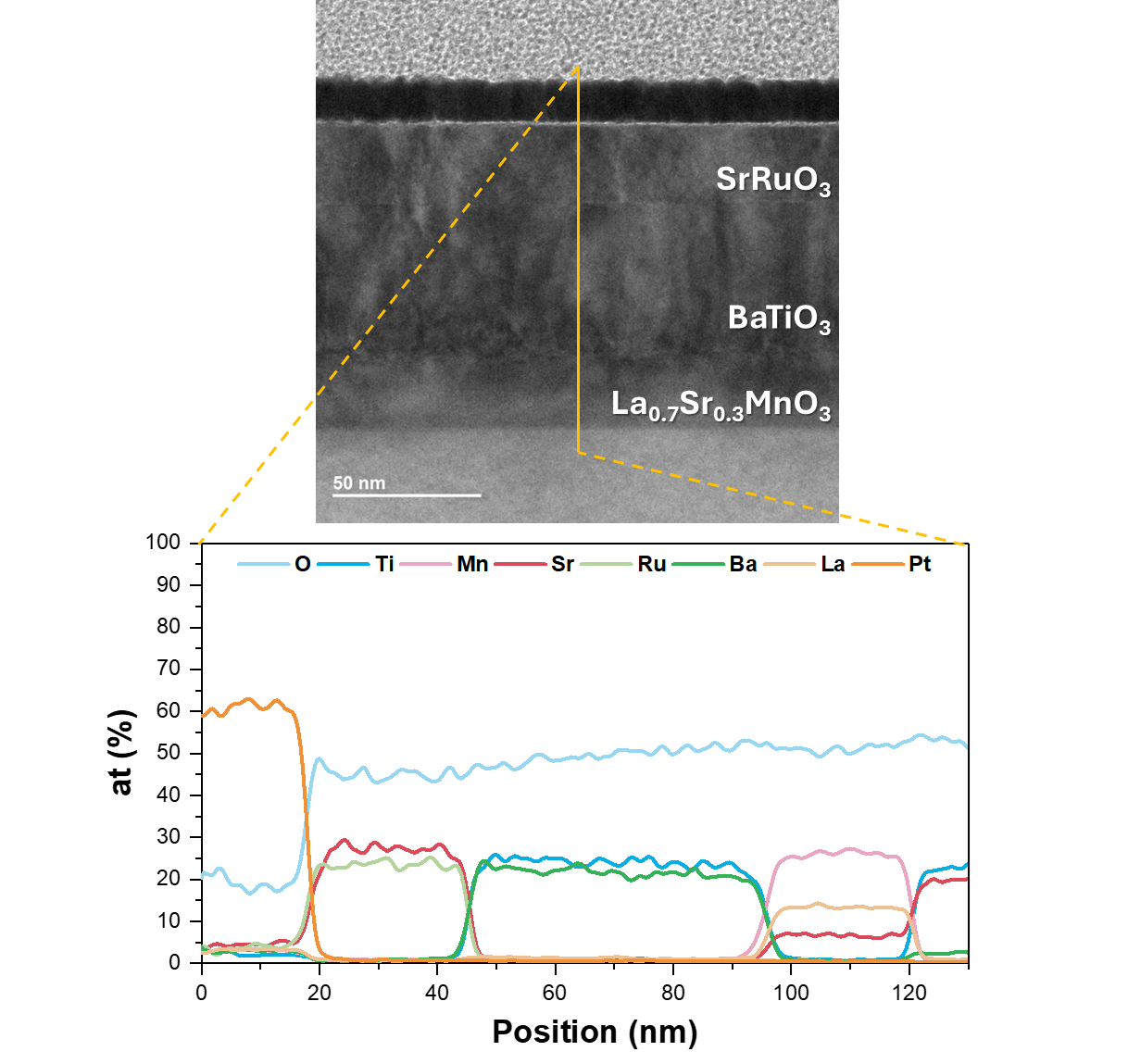
**

Cross-sectional TEM image and corresponding EDS elemental line profiles for the SrRuO_3_/BaTiO_3_/La_0.7_Sr_0.3_MnO_3_ heterostructure. The TEM image highlights the well-defined layered structure and the interfaces, while the EDS profiles show the elemental distribution across the interfaces. The results confirm the absence of interdiffusion and concentration gradients, indicating sharp and chemically distinct interfaces.

**S11.** **Imprint in fluorite-based ferroelectrics, Hf_0.5_Zr_0.5_O_2_.**


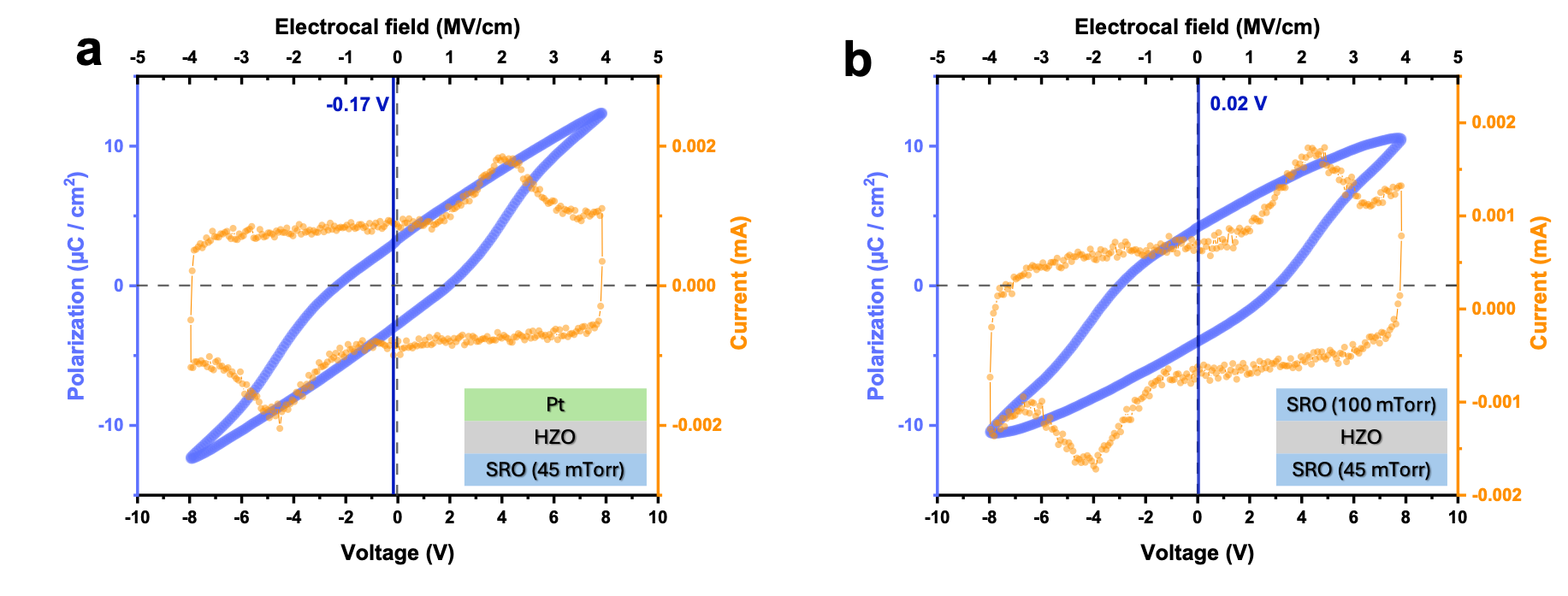


**a,** P-V and I-V hysteresis of asymmetric electrode device of Hf_0.5_Zr_0.5_O_2_. **b,** P-V and I-V hysteresis of symmetric electrode device of Hf_0.5_Zr_0.5_O_2._

**S12. Imprint in fluorite-based ferroelectric devices.**

**
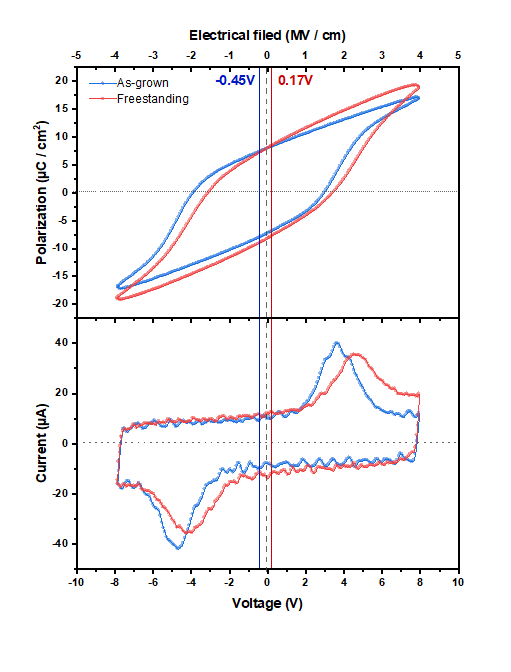
**

This work function theory also applies to fluorite-based ferroelectrics such as Hf_0.5_Zr_0.5_O_2_. Hf_0.5_Zr_0.5_O_2_ is selective regarding its bottom electrode, with the epitaxial polar phase often stabilizing only on specific electrodes, most commonly reported on LSMO. This limitation restricts the potential for improving imprint by minimizing work function differences. However, the freestanding technique allows for the easy transfer of freestanding Hf_0.5_Zr_0.5_O_2_membranes onto suitable bottom electrodes, thereby reducing the built-in voltage and imprint. In the following case, we replaced the Hf_0.5_Zr_0.5_O_2_ membrane onto W/Si after the freestanding process, resulting in a V_offset_ change from -0.45V to +0.18 V.

**S13. Reliability of SrRuO_3_/BaTiO_3_/SrRuO_3_ device.**

**
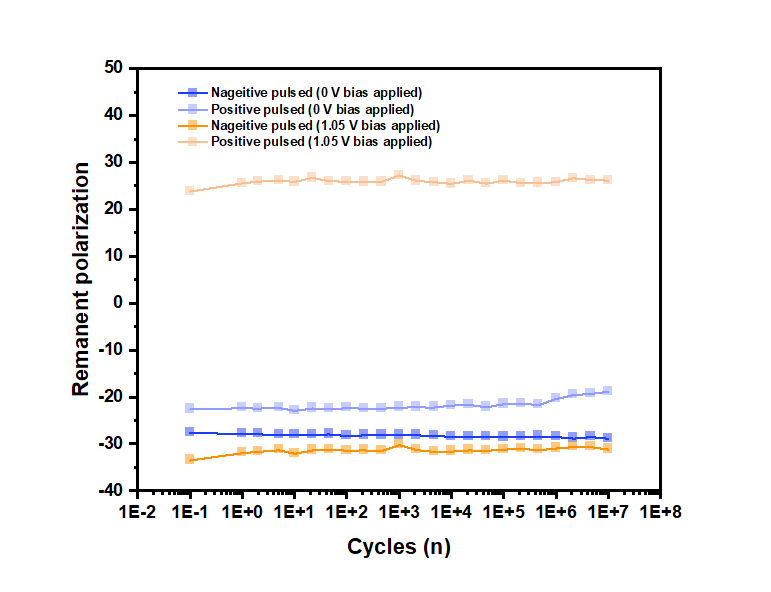
**

For a device with symmetrical top and bottom electrodes (SrRuO_3_/BaTiO_3_/SrRuO_3_) a dc bias of 1.05 V must be applied during electrical cycling. Without this bias, only a single measurable polarization state is observed, as indicated by the light blue markers.

**S14. The dependence of LSMO crystallinity on different oxygen growth pressures.
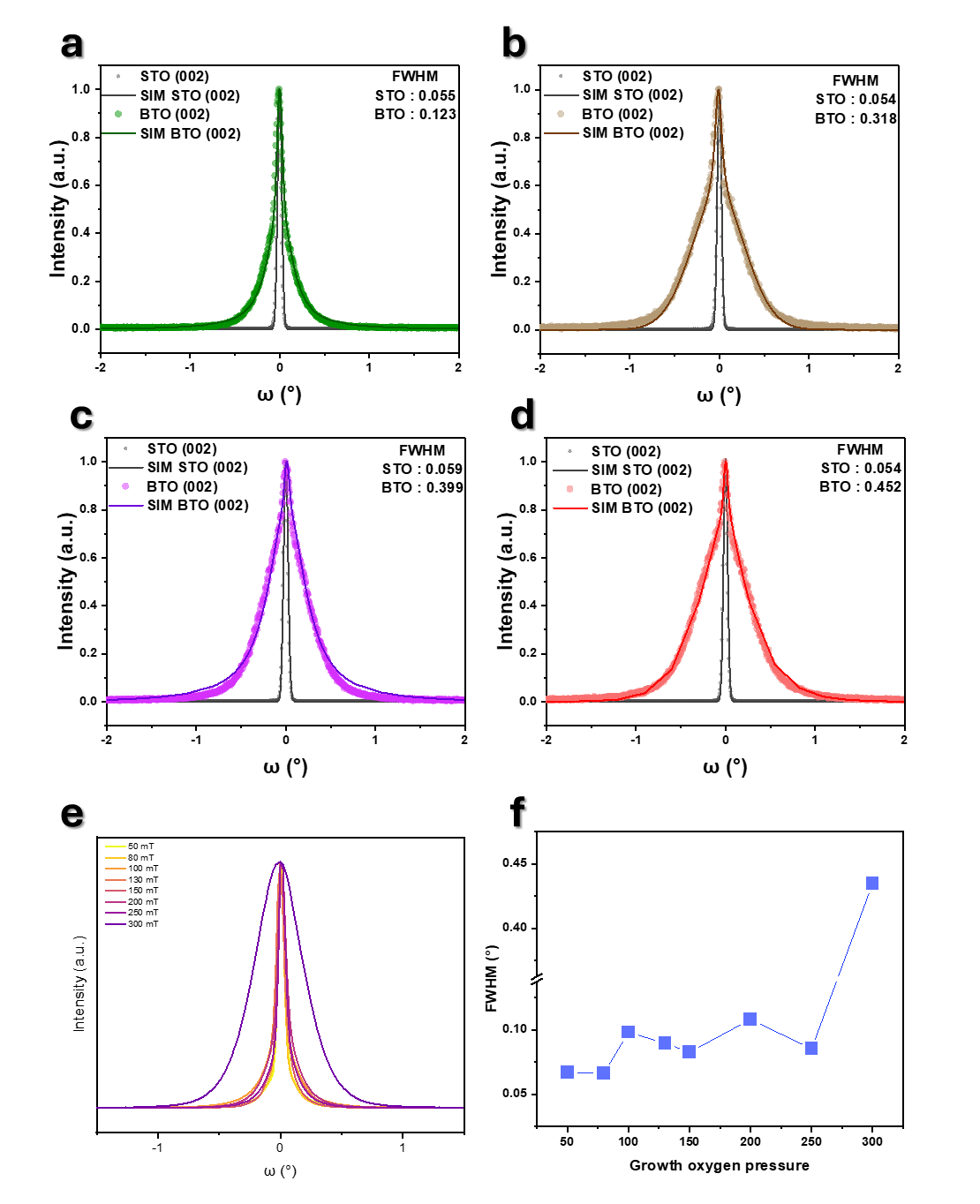
**

To assess the suitability of LSMO as a bottom electrode in a single-layer configuration, a 30 nm LSMO film was deposited under varying background oxygen pressures. Subsequently, ferroelectric (FE) layers, including BaTiO_3_ and BiFeO_3_, were grown on top. The crystallinity of the FE layers was evaluated using rocking curve measurements. Panels **a**, **b**, **c**, and **d** correspond to BaTiO_3_ films grown on LSMO layers prepared at background oxygen pressures of 150, 200, 250, and 300 mTorr, respectively. The results indicate that the crystallinity of BaTiO_3_ deteriorates as the full width at half maximum (FWHM) of the rocking curve broadens with increasing LSMO growth pressure. In panel **e**, the FWHM increases with higher LSMO growth pressures, suggesting a decline in the crystallinity of the BaTiO_3_ layer. Panel **f** provides the extracted FWHM values for BaTiO_3_, clearly illustrating the worsening crystallinity trend with increasing LSMO growth pressure.

**S15. Sensitivity of work function measurements to Ar-Ion sputtering time.**

**
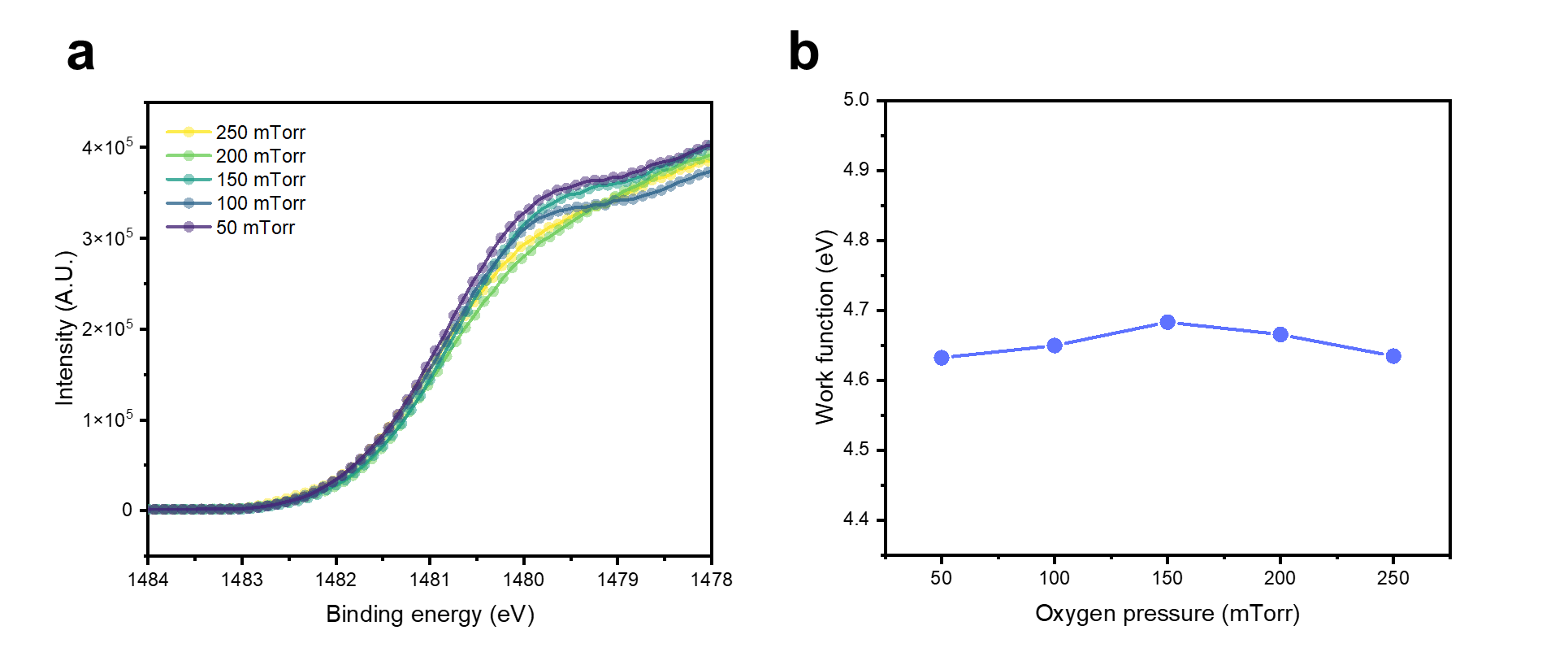
**

**a,** Work function measurements of LSMO thin films grown under varying oxygen pressures (50–250 mTorr), obtained via XPS after 30-second Ar-ion sputtering, indicating the contribution from near-bulk signal. **b**, Extracted work function values of LSMO thin films as a function of growth oxygen pressure.

**Reference**

1 Jacobs, R., Booske, J. & Morgan, D. Understanding and controlling the work function of perovskite oxides using density functional theory. *Advanced Functional Materials* **26**, 5471-5482 (2016).
